# Intracellular delivery of therapeutic antibodies into specific cells using antibody-peptide fusions

**DOI:** 10.1038/s41598-019-55091-0

**Published:** 2019-12-10

**Authors:** Julie Gaston, Nicolas Maestrali, Guilhem Lalle, Marie Gagnaire, Alessandro Masiero, Bruno Dumas, Tarik Dabdoubi, Katarina Radošević, Pierre-François Berne

**Affiliations:** 1 Yubsis, 4 rue Pierre Fontaine, 91000 Evry, France; 2 Sanofi R&D, Biologics Research, 13 Quai Jules Guesde, 94400 Vitry-sur-Seine, France; 3 Department of Immunology, Virology and Inflammation, UMR INSERM 1052, CNRS 5286, Centre Léon Bérard, Labex DEVweCAN, 693743 Lyon, France

**Keywords:** Biologics, Proteins

## Abstract

Because of their favorable properties as macromolecular drugs, antibodies are a very successful therapeutic modality for interfering with disease-relevant targets in the extracellular space or at the cell membrane. However, a large number of diseases involve cytosolic targets and designing antibodies able to efficiently reach intracellular compartments would expand the antibody-tractable conditions. Here, we genetically fused cell penetrating peptides (CPPs) at various positions to an antibody targeting cancer cells, evaluated the developability features of the resulting antibody-peptide fusions and the ability of selected constructs to reach the cytosol. We first determined positions in the IgG structure that were permissive to CPP incorporation without destabilizing the antibody. Fusing CPPs to the C-terminus of the light chain and either before or after the hinge had the least effect on antibody developability features. These constructs were further evaluated for cell penetration efficiency. Two out of five tested CPPs significantly enhanced antibody penetration into the cytosol, in particular when fused before or after the hinge. Finally, we demonstrate that specific antibody binding to the cell surface target is necessary for efficient cell penetration of the CPP-antibody fusions. This study provides a solid basis for further exploration of therapeutic antibodies for intracellular targets.

## Introduction

Over the years, antibodies have been established and are becoming increasingly important as therapeutic modality for serious diseases, such as rheumatoid arthritis and other autoimmune diseases^1–3^. Novel antibody therapies have revolutionized cancer treatment and have offered hope for many patients who previously had no effective treatment options for their condition^4,5^. The number of approved biologics is steadily increasing with 12 novel antibody therapeutics having gained approval in either the European Union or United States in 2018^6^, and seven biologics are currently among the 10 top-selling drugs worldwide^7^. The success of antibody therapies is due to the fact that antibodies bind very specifically to their target, like a lock and key, which makes them efficacious and safe, with low unpredictable side-effects. In addition, due to their large size, antibodies circulate for a long time in the human body and therefore can exert long term activity^8^.

Antibodies are an excellent therapeutic modality for easily accessible targets, such as secreted or cell-surface expressed molecules. The major limitation for further expanding the antibody target space lies in their inability to efficiently penetrate cells and reach the cytosol. It has been estimated that more than 20% of proteome is located inside cells, representing a large number of potentially interesting but antibody-inaccessible therapeutic targets (https://www.proteinatlas.org/humanproteome/cell/cytosol). These include oncogenic proteins, components of different signal transduction pathways, cell metabolism regulators and different enzymes^9^. The intracellular target space is currently restricted to small molecules, chemical entities with much less selectivity, shorter half-life and increased chance for adverse effects as compared to antibodies. Finding solutions to reach intracellular targets efficiently with antibodies would thus pave the way to a large range of new antibody therapeutic opportunities.

The major obstacle for an antibody to exercise its activity inside the cell is crossing the cell membrane and/or the endosomal membrane upon receptor mediated internalization. There have been reports on antibodies with intrinsic capability to internalize. A number of studies have been published on two anti-DNA antibodies, called 3E10 and 3D8, originating from autoimmune diseases and able to internalize and localize inside the nucleus and cytosol, respectively^10,11^. In the case of 3E10, cell penetration and nuclear delivery was shown to be mediated by the ENT2 nucleoside salvage pathway^12^. In this case, the penetrating antibody, 3E10 ScFv, has been used as vehicle to deliver an active moiety inside cells, such as p53^13^, or an anti-mdm2 ScFv^14^. In the case of 3D8, cytosolic delivery was shown to be related to a specific antibody sequence within the light chain CDR3, which mediated endosomal escape through a conformational change upon acidification in the endosome^15^. When this light chain was combined with a heavy chain specific for oncogenic protein ras, it was shown to reduce proliferation of ras-dependent tumor cells^16^. This elegant approach shows that a short sequence within an antibody may be sufficient to promote cytosolic delivery. However, since this cytosol-penetrating sequence is within a CDR, a complex engineering approach may be necessary in order to facilitate antibody penetration while not impairing the affinity and specificity of the parental antibody. Furthermore, in this approach, fusion of an integrin-binding peptide to the antibody was necessary to enable target cell specificity and enhance efficacy of uptake, further complicating the design of potential therapeutic antibody.

Yet another approach to get large molecules into the cells is using cell-penetrating peptides (CPPs) as a cross-membrane transport vehicle. This category of small peptides (ranging from 5 to 30 amino acids), has become an interesting tool for intracellular delivery, as they have demonstrated their ability to cross cellular membranes^17–19^.

So far, CPPs linked to proteins by either non-covalent linkage, or chemical or peptide covalent linkage have been described. They have been fused to several types of proteins, DNA or RNA, with encouraging results and generating hope for therapeutic potential^20^. However, chemically and randomly fusing CPPs results in a difficult to characterize and optimize mixture and hampers efficient product development. In addition, chemical conjugation can impair specificity to the intracellular target^21^. In a recent study, an anti-HBV IgG with a C-terminally fused TAT peptide on the heavy chain was shown to suppress Hepatitis B virus intracellularly^18^. In this case, like with the chemical conjugation examples, there was a lack of targeting to specific cells, which has disadvantage for dosing and safety, since the fusion protein can end up in any cell and not only in the cells of choice. Taken together, previous attempts suggest that CPP-grafted antibodies with specificity to an intracellular target may also need specific binding to a cell-surface antigen to achieve efficient cell penetration in a cell-type specific manner.

In the current study, we investigated the feasibility of CPPs genetically fused to full-length IgG to serve as a shuttle for targeting the antibody into the cytosol of specific cells. Instead of focusing on the antibody activity inside cells, we chose to focus first on efficient cell penetration of specific cells, which is considered the major obstacle, and to postpone the choice of an intracellular target for future work. We explored fusing CPPs to different positions on an IgG, with specific attention to “developability” features of the final fusion constructs, which are relevant for therapeutic product development. As a model system we have used an antibody (Ab) specific for carcinoembryonic antigen-related cell adhesion molecule 5 (CEACAM5), a tumor associated antigen expressed at the surface of tumor cells^22^, in a combination with a set of CPPs with different biochemical characteristics. Our results demonstrate (i) that certain CPPs can facilitate antibody penetration into the cytosol, (ii) the importance of selecting the right fusion position on IgG and (iii) the importance of specific binding of the antibody to the cell surface for efficient intracellular penetration.

## Results

### Developability of CPP-Ab fusion constructs

**Figure 1:**
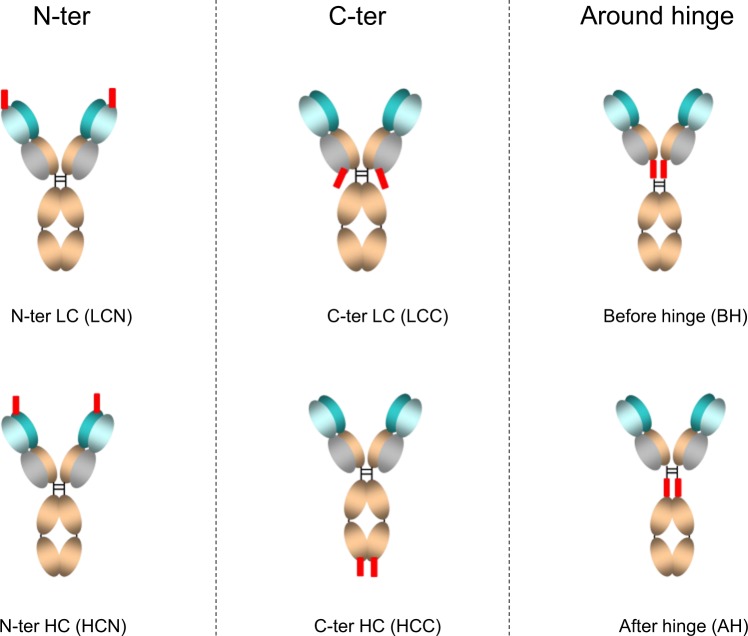
Schematic representation of the different sites where the CPPs (red rectangles) were inserted in the antibody. Four positions consist in introducing CPP at the N- or C-terminus of the heavy chain (HC) or light chain (LC). Two other positions exploit the hinge: “before hinge” represents the insertion of the CPP before the Cys-Pro-Pro-Cys-Pro (CPPCP) amino acids with a Gly-Ser (GS) linker after the CPP sequence; “after hinge” represents the insertion of the CPP after the CPPCP amino acid with GS and Gly (G) amino acids inserted before and after the CPP, respectively.

Envisioning possible future therapeutic applications, we have set the appropriate Chemistry, Manufacturing and Control (CMC) features (“developability”) of the fusion constructs as important selection criteria. Therefore, we first evaluated the impact of fusing a CPP to six different positions in the IgG backbone on the developability characteristics (Fig. 1). We evaluated fusing the CPP to the N- or C-terminus of either the heavy or light chain, hypothesizing that the CPP could be more accessible to efficiently interact with the cell- or endosomal membranes and facilitate penetration. Furthermore, as the hinge is a flexible domain and this position has been described as allowing introduction of a scFv^23^, we evaluated the impact of inserting a CPP directly before the hinge domain (“before hinge”) as well as directly after the hinge domain (“after hinge”).

Based on promising published results, we selected a set of cell-penetrating peptides of different length and biochemical features (Table 1). In order to select the best positions for fusing the CPPs, we first generated six CEACAM5 antibodies fused with two of the peptides (TAT^24,25^ and Pep-1^26^) to each of the six positions depicted in Fig. 1. Pep-1 was chosen as the first amphipathic CPP to be tested as it has previously been chemically bound to anti-LAMP1 and anti-beta-actin and demonstrated to facilitate antibody penetration into cells^26^. Tat is the first cationic CPP that has demonstrated capacity to introduce diverse types of proteins into cells, such β-galactosidase, anti-BCL2-scFv and more recently antibodies^18,24,25,27^. The developability features (and acceptance criteria) for the final constructs included: antibody expression after 7 days of culture (>50 mg/L), SEC profile after affinity chromatography (1 major single peak), purity after affinity and SEC chromatographies (>90%), purification yield (preferred >60%, acceptable 40%), experimental mass by liquid chromatography-mass spectrometry (LC-MS; equal to theoretical mass). The TAT constructs were either not expressed or were truncated within TAT sequence whatever the insertion position and, therefore, could not be further characterized (Table 2). In contrast, Pep-1 fusions could be purified, although significant differences were observed in developability features depending on the Pep-1 insertion position (Table 2). In the case of N-terminal insertion in the heavy chain (HCN) or light chain (LCN), no antibody fusion was detected in cell culture. Fusion of Pep-1 to the C-terminus of the heavy chain (HCC) resulted in too low yield and/or purity, as demonstrated by the presence of several peaks on SEC profile and non-expected forms in mass spectrometry. CPP-antibodies with fusion in the C-terminus of the light chain (LCC), before hinge (BH) and after hinge (AH) fulfilled all developability criteria, although some differences in developability were observed. In order to ensure that CPP insertion does not impact antibody stability, the thermal stability was determined using DSF, showing no significant difference between the different constructs and the control antibody without CPP (Table 3). For these 3 positions, we also confirmed that the binding affinity to CEACAM5 was not significantly affected, as shown by a less than 2-fold variation, as compared to the control (Supplementary Table S1).

**Table 1:** Characteristics of the selected Cell Penetrating Peptides (CPPs).

Name	CPP length	Properties	Net charge per CPP	Sequence	Ref.
Pep-1	21	Amphipathic	3	KETWWETWWTEWSQPKKKRKV	^26^
TAT	11	Cationic	8	YGRKKRRQRRR	^24,25^
PEPth	12	Cationic	5	VKKKKIKAEIKI	^28^
aurein 1.2	13	Amphipathic	1	GLFDIIKKIAESF	^29^
MTS	17	Hydrophobic	0	KGEGAAVLLPVLLAAPG	^19,30^
GFWFG	5	Hydrophobic	0	GFWFG	^31^

**Table 2:** Developability of CPP-Abs depending on selected insertion positions.

CPP	Position	Ab concentration in culture supernatant (mg/L)	SEC after affinity purification	Purity after 2 step purification (%)	Purification yield (%)	LC-MS
no	Not applicable	141	1 peak	97	80	Expected mass
Pep-1	N-ter LC (LCN)	2	nd	nd	nd	nd
Pep-1	C-ter LC (LCC)	145	1 peak	96	80	Expected mass
Pep-1	N-ter HC (HCN)	1	nd	nd	nd	nd
Pep-1	C-ter HC (HCC)	113	6 peaks	94	27	Expected mass + others
Pep-1	before hinge (BH)	132	1 peak	97	63	Expected mass
Pep-1	after hinge (AH)	120	1 peak	95	43	Expected mass
TAT	N-ter LC (LCN)	4	nd	nd	nd	nd
TAT	C-ter LC (LCC)	174	2 peaks	74	43	truncated LC in TAT sequence
TAT	N-ter HC (HCN)	5	nd	nd	nd	nd
TAT	C-ter HC (HCC)	190	2 peaks	88	20	truncated LC in TAT sequence
TAT	before hinge (BH)	156	6 peaks	75	26	truncated HC in TAT sequence
TAT	after hinge (AH)	156	3 peaks	67	41	truncated HC in TAT sequence

Nd: not determined. SEC: size exclusion chromatography.

**Table 3:** Thermal stability of CPP-Abs.

CPP	Position	Tagg	Tm(Fc)	Tm (Fab)
no	Not applicable	82,3 °C	70,9 °C	83,5 °C
Pep-1	C-ter LC (LCC)	81,0 °C	70,5 °C	83,3 °C
Pep-1	before hinge (BH)	80,9 °C	68,9 °C	83,0 °C
Pep-1	after hinge (AH)	81,2 °C	68,3 °C	82,9 °C

Tagg: aggregation temperature, Tm(Fc): melting temperature of Fc domain, Tm(Fab): melting temperature of Fab domain.

In the next step, we fused four additional CPPs at the three identified positions for further evaluation and characterization of their intracellular penetration capability: PEPth^28^, aurein 1.2^29^, MTS^19,30^, and GFWFG^31^ (Table 1). All constructs included a cyto-Tag (GFP11-SBP2) for cytosolic detection by GFP complementation assay, fused to the C-terminus of the heavy chain via a (G4S)3 linker^32^. The cytotag GFP11-SBP2 consisted of Green Fluorescent Protein (GFP) 11 (RDHMVLHEYVNAAGIT), followed by (G4S)3 linker and the Streptavidin Binding Peptide (SBP) 2 (GHVVEGLAGELEQLRARLEHHPQG). Antibodies fused to CPPs were abbreviated as CPP-Abs (cell penetrating peptide antibodies) and are named based on the inserted CPP and the insertion position.

The fusion of CPPs within chosen positions was well tolerated and all fusion constructs fulfilled the developability criteria, except for PEPth-LCC and GFWFG-AH, which reached 87% purity (instead of 90%) and showed a purification yield of 38% (instead of 40%), respectively (Table 4). In two cases (Pep-1-BH and PEPth-LCC), two peaks were detected by size-exclusion chromatography (SEC) analysis after affinity purification. However, the first peak, corresponding to a fraction of aggregated protein, could be easily removed during the purification process yielding a final purity ranging from 90% to 99%. Importantly, the introduced CPPs did not affect the binding affinity for CEACAM5 of the CPP-Abs (Supplementary Table S2).

**Table 4:** Developability of different CPP-Abs with cytosolic tag.

Fusion construct	CPP	Position	Cyto-solic tag	Ab concentration in culture supernatant (mg/L)	SEC after affinity purification	Purity after 2 step purification (%)	Purification yield (%)	LC-MS
Ab-ctrl1	no	NA	−	141	1 peak	97	80	Expected mass
Ab-ctrl2	no	NA	+	123	1 peak	96	85	Expected mass
Pep-1-LCC	Pep-1	C-ter LC	+	77	1 peak	93	43	Expected mass
Pep-1-BH	Pep-1	before hinge	+	96	2 peaks	95	40	Expected mass
Pep-1-AH	Pep-1	after hinge	+	86	1 peak	94	48	Expected mass
PEPth-LCC	PEPth	C-ter LC	+	59	2 peaks	87	59	Expected mass
PEPth-BH	PEPth	before hinge	+	80	1 peak	95	64	Expected mass
PEpth-AH	PEPth	after hinge	+	79	1 peak	90	67	Expected mass
Aurein-LCC	aurein 1.2	C-ter LC	+	110	1 peak	97	64	Expected mass
Aurein-BH	aurein 1.2	before hinge	+	103	1 peak	99	63	Expected mass
Aurein-AH	aurein 1.2	after hinge	+	117	1 peak	99	54	Expected mass
MTS-LCC	MTS	C-ter LC	+	100	1 peak	95	61	Expected mass
MTS-BH	MTS	before hinge	+	109	1 peak	99	60	Expected mass
MTS-AH	MTS	after hinge	+	118	1 peak	90	53	Expected mass
GFWFG-LCC	GFWFG	C-ter LC	+	121	1 peak	94	43	Expected mass
GFWFG-BH	GFWFG	before hinge	+	100	1 peak	99	54	Expected mass
GFWFG-AH	GFWFG	after hinge	+	107	1 peak	92	38	Expected mass

### Intracellular penetration of antibody-peptide fusion constructs

It is experimentally very difficult to properly distinguish molecules released in cytosol from those trapped in the endocytic vesicles or other organelles, and quantify them accurately using imaging techniques^33,34^ and subcellular fractionation^35^. In particular cell fixation has been exemplified to induce re-localization of proteins into different cell compartments and thus experimental artefacts^36^. Therefore, we used a simple and direct assay for quantitative assessment of the amount of cytosolic molecules, based on a split GFP fluorescence complementation assay^32^. When the large fragment of GFP (GFP1-10SA) encounters the small fragment (GFP11-SBP2, called cyto-Tag) fused to the C-terminus of the heavy chain, the two GFP fragments complement each other and GFP fluorescence can be detected (see Methods for further details). Before analyzing the cell-penetrating ability of the CPP-antibodies, we validated the functionality, kinetics of response and sensitivity of the cyto-Tag in the complementation assay as follows. First, we confirmed that all CPP-Abs with cyto-Tag are equivalent in their ability to complement, in a dose- and time-dependent manner, with GFP1-10SA in the lysate of HEK293FS cells that had been transfected with GFP1-10SA expressing plasmid (Supplementary Figure S1a). Ab-ctrl1, which has no cyto-Tag, does not produce GFP. In contrast, Ab-ctrl2 and all the CPP-Abs, which carry the cyto-Tag, are able to complement and generate GFP. This experiment has been done for every construct with similar result, but only two representative samples are shown for clarity. The time-dependency of fluorescence, reaching a maximum at 8 hours, can be explained by the time needed for maturation of the GFP fluorophore^37^. Second, using a fixed amount of antibody (0.5 μg) and a fixed incubation time (8 hours, which corresponds to the beginning of the fluorescence plateau) we demonstrated that all tagged antibody-peptide constructs have a similar efficiency in the GFP1-10SA complementation (Supplementary Figure S1b). And, finally, we demonstrated using flow cytometry that the CPP-Abs also complement GFP1-10SA intracellularly when the fusion proteins are electroporated into GFP1-10SA-transfected HEK293FS cells (Supplementary Figure S1c). These results confirm the validity of cyto-Tag complementation assay and demonstrate that this assay can be used for unbiased comparison of cytosolic localization of the CPP-Abs.

Next, we screened all tagged CEACAM5 antibody-peptide constructs for their ability to penetrate cells and reach the cytosol using a LS174T colon carcinoma cell line, which expresses CEACAM5 at the cell surface. Cells were transiently transfected with a plasmid expressing GFP1-10SA. After 24 hours, purified fusion constructs or control antibodies were added to the culture medium at 2 µM concentration and cells were incubated for 24 hours. Twenty four hours has been selected for antibody incubation based on testing several time points (between 6 and 48 hours, data not shown) since at this time point a balance seems to be reached between different intracellular processes (i.e. the kinetics of the antibody reaching the cytosol, associating with the complementary GFP1-10SA fragment and developing the fluorophore on one hand, and the TRIM21-related degradation kinetics on the other hand^38^). The samples were analyzed using flow cytometry for the percentage of GFP positive cells, mean fluorescence intensity (MFI) of GFP positive cells and cell viability.

**Figure 2:**
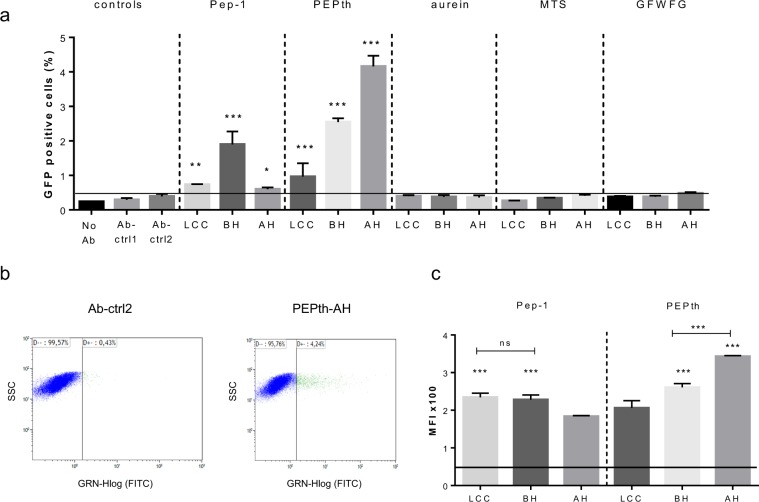
Screening of different CPP-Abs for cytosol penetration. LS174T cells were transfected with GFP1-10SA and subsequently incubated with CPP-antibodies at 2 µM for 24 hours before being analyzed by FACS. (**a**) Percentage of GFP-positive cells. The horizontal line represents the percentage of GFP positive cells with Ab-ctrl2 (background). For statistical analysis, the reference sample is Ab-ctrl2. *P < 0.02, **P < 0.002, ***P < 0.0001. (**b**) Dot plot of 2 samples as examples: Ab-ctrl2 and PEPth-AH: x-axis, labeled as FITC, represents GFP fluorescence intensity, and y-axis, SSC side-scattered light, represents granularity. (**c**) Mean fluorescence intensity (MFI) of GFP positive cells. The horizontal line represents cells autofluorescence (background). One-way ANOVA with Fisher’s LSD comparison; samples are compared with the lowest MFI samples (respectively AH and LCC for Pep-1 and PEPth), ***P < 0.0001.

As can be seen in Fig. 2, control antibodies without Cyto-Tag (Ab-ctrl1) and with Cyto-Tag but without CPP (Ab-ctrl2), gave less than 0.5% positive cells. Six CPP-Abs, all with peptides Pep-1 or PEPth, significantly increased the percentage of GFP-positive cells, with the PEPth-AH fusion construct being the most efficient (Fig. 2a,b). For the CPP-Abs that successfully reached the cytosol of the cells, the MFI of GFP-positive cell population was calculated and was high above background level given by cells autofluorescence. PEPth-AH, which penetrated a higher percentage of the cells than PEPth-BH, also resulted in a higher MFI, meaning that relatively more PEPth-AH than PEPth-BH molecules entered the cells. In contrast, in the constructs with Pep-1 as CPP, Pep-1-LCC induces less GFP positive cells than Pep-1-BH but they have a similar MFI (Fig. 2c). We concluded that the number of GFP positive cells and the mean fluorescence intensity might not necessarily correlate, depending on CPP insertion site and type of CPP, and suggesting possibly different mechanisms of penetration. Using a viability marker during FACS analysis, we confirmed comparable cell viability for all samples (Supplementary Figure S2).

**Figure 3:**
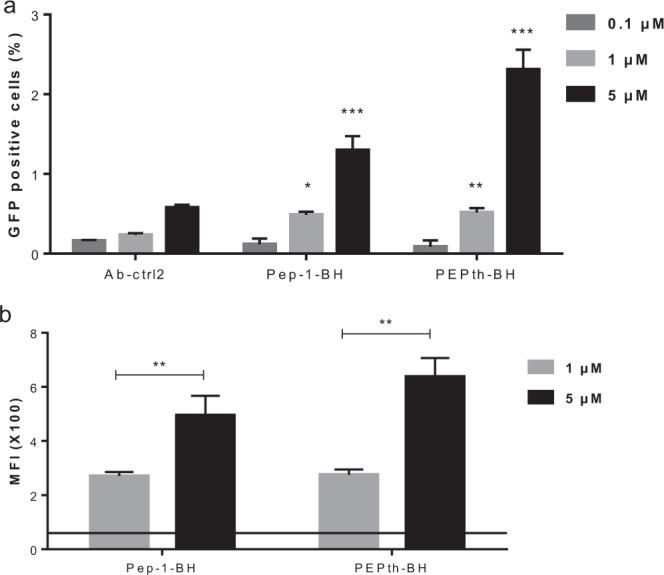
Dose-dependency of cytosolic delivery of Pep-1-BH and PEPth-BH. LS174T cells were transiently transfected with GFP1-10SA expressing plasmid, then incubated with CPP-Abs at 0.1, 1 and 5 µM for 24 hours before analysis by FACS. (**a**) Percentage of GFP-positive cells. Statistical analysis was used to compare CPP-Abs to Ab-ctrl2. *P < 0.02, **P < 0.007, ***P < 0.0001. (**b**) Mean Fluorescence Intensity of GFP-positive cells of Pep-1-BH and PEPth-BH. The horizontal line represents cells autofluorescence (background). Statistical analysis was used to compare 1 µM and 5 µM conditions. **P < 0.01.

For further studies, we selected Pep-1-BH and PEPth-BH, which contain two different CPPs (Pep-1 and PEPth, respectively) introduced at the same position (before hinge), evaluated the dose dependency of the internalization and quantified the number of internalized antibody molecules. As can be seen in Fig. 3A, specific internalization could be detected after 24 h incubation with 1 µM CPP-Ab fusion constructs and was even more significant in samples with 5 µM CPP-Ab. In both cases, an increased concentration of CPP-Ab led to an increase of mean fluorescence intensity in positive cells (Fig. 3B). Thus, increasing the extracellular concentration of CPP-Abs led to both a higher percentage of cells with intracellular antibody and a higher amount of intracellular antibody per positive cell.

In order to quantify the amount of antibodies in the cytosol of the GFP-positive cell populations, we first established a calibration coefficient between the number of GFP molecules and the mean fluorescence intensity using GFP calibration beads (beads loaded with known numbers of GFP molecules, 10^4^ to 10^8^) that were analyzed by FACS (Supplementary Figure S3 and Supplementary Table S3). Cells incubated with 1 µM and 5 µM CPP-Ab constructs were analyzed using FACS and the number of molecules per cell was calculated according to this calibration, as described in Methods (Table 5). The cytosolic concentrations of Pep-1-BH and PEPth-BH applied at 5 µM reached 643 nM and 851 nM, respectively.

**Table 5:** Quantification of the amount of CPP-Abs in the cytosol of GFP-positive cells.

	1 µM			5 µM		
	MFI values (×100)	Number of intracellular antibody molecules ×10^5^	nM per cell	MFI values (×100)	Number of intracellular antibody molecules ×10^5^	nM per cell
Pep-1-BH	2.7 ± 0.2	1.7 ± 0.1	317 ± 20	4.0 ± 0.7	3.6 ± 0.6	643 ± 105
PEPth-BH	2.7 ± 0.1	1.8 ± 0.1	324 ± 28	6.4 ± 0.7	4.8 ± 0.5	851 ± 101

The values represent mean ± SD of at least three independent experiments.

### Influence of antibody binding to CEACAM5 on cell penetration

**Figure 4:**
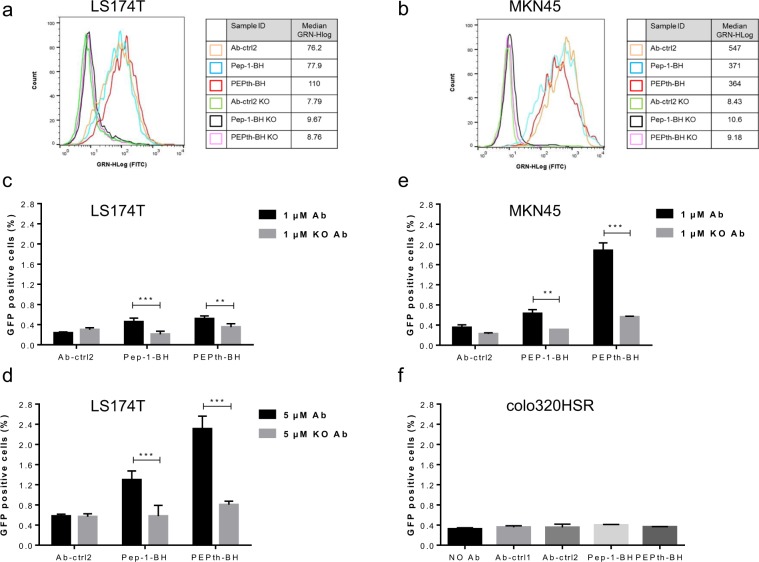
Binding of CPP-Abs to CEACAM5 is necessary for intracellular penetration. Histogram for LS174T (**a**) and MKN45 (**b**) cells after incubation on ice for 45 min with each CPP-Ab. LS174T cells were transiently transfected with GFP1-10SA, then incubated with CPP-Abs or their KO variant (not binding CEACAM5) for 24 hours before being analyzed by FACS (**c**: 1 µM, **d**: 5 µM). MKN45 cells were transiently transfected with GFP1-10SA, then incubated with CPP-Abs or their KO variant (not binding CEACAM5) for 24 hours before being analyzed by FACS (**e**). Colo320HSR cells, which do not express CEACAM5, were transiently transfected with GFP1-10SA, then incubated with CPP-Abs at 1 μM during 24 hours before FACS analysis (**f**). **P < 0.004, ***P < 0.0002.

As CPPs may interact with any kind of cell membrane, we evaluated if the CPP moiety in our CPP-Abs is able to mediate the binding to cancer cells. For this purpose, we fused Pep-1 and PEPth to a control antibody which contains mutations in the CDRs that abolish the binding to CEACAM5 (CEACAM5-KO). These CPP-KOAbs bind neither to LS174T nor to MKN45 (a gastric cancer cell line which also expresses CEACAM5), as determined by flow cytometry, demonstrating that the CPP alone does not mediate non-specific interaction with the cell surface (Fig. 4A,B). The CPP-Abs were subsequently tested for their ability to penetrate LS174T and MKN45 cells using the split GFP complementation assay. With both cell lines we could detect intracellular penetration of CPP-Abs, and in both cases PEPth-BH seemed to be more readily internalized (Fig. 4C–E). Interestingly, penetration of MNK45 cells seemed more efficient than of LS174T cells since 1 μM CPP-Ab on MKN45 yielded the same percentage of GFP positive cells as 5 μM CPP-Ab on LS174T (Fig. 4D,E). This is probably due to the fact that MNK45 cells express a higher level of CEACAM5 than LS174T. Abolishing the specific binding of the antibody to cells by knocking out the CEACAM binding site (CAECAM5-KO) completely abolished the delivery of the fusion constructs into the cytosol (KO Ab in Fig. 4C–E). The dependence of the intracellular penetration on binding to the cell surface receptor was further corroborated using colo320HSR, a cell line which does not express CEACAM5. When used as the target cell in the complementation assay, cytosolic delivery of neither Pep-1-BH nor PEPth-BH antibody constructs was detected (Fig. 4F).

Taken together, these results provide strong evidence that CPP-Abs can be delivered into cytosol and that binding of the CPP-Ab to a specific cell surface receptor is necessary for efficient intracellular delivery.

## Discussion

Our work demonstrates that certain cell penetrating peptides can enhance intracellular penetration of antibodies, leading to significant concentration of the antibody in the cytosol. To achieve this, we first selected “permissive” positions in the antibody backbone, i.e. positions in which insertion of the peptide neither alters the developability features nor the binding specificity of the antibody. We then identified CPP sequences that enhanced cytosolic localization. Finally, we demonstrated that specific antibody binding to a cell surface receptor is necessary for achieving a significant antibody concentration in cytosol.

In our attempts to explore various genetic fusions between CPPs and an antibody, we looked carefully at the potential impact on antibody developability. Therapeutic antibody engineering needs to take this parameter into account very early in construct selection, since developability issues discovered at late stage usually result in unexpected development delays, costs, or even in project termination. In addition, previous literature suggested that CPP sequences, due to their intrinsic propensity to interact with membranes, could interfere with the mammalian secretion pathway. Indeed, during the screening of positions, we were unsuccessful in generating a fusion of TAT peptide, a well-known CPP^24,25^, with an IgG. In our case, TAT insertion in the IgG sequence resulted in either loss of expression or proteolytic degradation and we hypothesized that it could be due to interference of the TAT sequence with the secretion pathway and/or recognition of TAT sequence by furin-like proteases^39^. Noteworthy, a construct where TAT is grafted at the C-terminus of the heavy chain has been described by others^18^, but no expression issues were reported. The two studies differ in the cell type used for production (HEK293FS versus CHO) and, in addition, we fused the CPP directly to the last amino acid of the antibody sequence, whereas a short linker was added in the mentioned study, which could have influenced the construct stability.

Fusions with Pep-1 at the N-terminus of either of the antibody chains also resulted in expression issues and we speculate that this may be related to interference with secretion. However, we have identified three other locations in the antibody that were permissive for insertion of PEP-1, and they were also compatible with four other CPPs. Since these positions are in the constant domains, they can potentially be applied to any antibody. Since in our study we only used CEACAM5 antibody for fusing the CPP, additional work is needed to confirm that the same positions are equally permissive when applied to IgGs with different variable domains. Ultimately, it was important to identify few positions that tolerate production of CPP-Ab fusions, since we hypothesized that the efficiency of CPPs in terms of cell penetration may depend not only on the type of CPP but also on its localization within the antibody architecture. For example, when the CPP is inserted in the hinge, two CPP fragments would also be in close vicinity to each other, potentially facilitating more avid interaction with the membrane. It is interesting that the most efficient CPP-Abs in our study indeed had CPP fused in the hinge region.

The exact mechanism(s) of cell-penetration for different CPP-Abs still needs to be determined, and it may depend both on the type of CPP and the insertion site, as suggested by our results. Due to their cationic, amphipathic or hydrophobic nature, CPPs can disturb membrane bilayers and have been described to facilitate the uptake of DNA, RNA or proteins into cells and to promote endosomal escape^40^. Our study emphasizes that not all the CPPs are equally efficient. CPP-Abs based on Pep-1 and PEPth insertions showed the most significant efficiency of cytosol penetration into LS174T cell line and were confirmed with MKN45, another CEACAM5 expressing cell line. The most efficient peptide, PEPth, contains five net positive charges and it is possible that electrostatic interaction with the membrane is part of the mechanism of action. Pep-1 is an amphipathic CPP: a first part is hydrophobic and contains several tryptophan residues (W) that can be involved in membrane destabilization processes^25^, and a second part is cationic with lysine and arginine residues. Either or both features may possibly contribute to the cell penetration mechanism of antibody fused to Pep-1. In contrast, Aurein 1.2, MTS and GFWFG, that contain few or no positive charges, were not able to induce significant cell penetration. These results are in contrast to previous studies showing cell penetration with GFWFG^31^ and MTS peptides^19,30^. However, in the study with GFWFG, the cargo was GFP11, which is much smaller than an IgG^31^. As for MTS, antibodies were tested and successfully penetrated the cells but this was achieved using a random chemical conjugation with MTS peptide at 10 to 50 fold molar excess and without quantification of the final the peptide-antibody ratio^19,30^.

In our cell-penetration experiments, the percentage of GFP positive cells, representing cells in which enough antibodies are delivered into the cytosol to enable detection, is in the range of 5%. This percentage is probably an underestimation of intracellular penetration because only a fraction of the cell population efficiently expresses GFP1-10SA upon transfection (only about ~30% of cells for LS174T and ~20% of cells for MKN45), which is the prerequisite for the complementation and detection of CPP-Ab. In addition, TRIM21-mediated degradation of cytosolic IgG fusions could also lead to decrease of measurable signal and using Fc variant with abolished binding to TRIM21 would be an approach to test this hypothesis. Another and more speculative reason for relatively modest percentage of the GFP positive cells could be that cells are in different metabolic states and therefore differently responsive to antibody uptake or release into the cytosol. More sensitive, preferably target-related functional assays are needed to more efficiently detect cells with intact antibody in cytosol.

Importantly, while the GFP-positive population remains modest in percentage, the average concentration of cytosol-localized antibody inside those cells was shown to be in the 500 nM range, which represents about 10% of the extracellular concentration. Considering that intracellular concentration of the most abundant cytosolic proteins is in a hundreds nM range^41^, this antibody concentration is probably sufficient to neutralize a large number of intracellular targets. The cell penetrating anti-ras antibody described by Shin *et al*.^16^ was shown to efficiently reduce cell proliferation *in vitro* at 2 and 10 µM extracellular antibody concentration, yielding around 200 nM antibody concentration in the cytosol. *In vivo* studies using anti-ras or an anti-HBV antibody demonstrated the limitations of these constructs by showing that the specificity towards the targeted cells needs to be improved^18^. With our strategy, using target cell-specific antibody as the basis module for fusing CPP and, as we demonstrate, being the key component of the approach, the desired targeting to specific cells is assured while no uptake by irrelevant cells is taking place.

In conclusion, with this study we have established a solid basis for further developing exciting next generation of antibody therapeutics targeting intracellular targets, and would like to end by suggesting directions for future work. A first step would be to further optimize our most efficient CPP-Ab compounds, Pep-1-BH or PEPth-BH, by additional modifications of the CPP sequences. In parallel, it should be evaluated whether the same CPP insertions can also promote cytosolic delivery of other, different antibodies. Finally, introducing functionalizing CPPs into a bispecific IgG antibody, a novel class of biotherapeutics, with one antibody arm enabling specific cell targeting through surface antigen binding, and a second arm directed against an intracellular target, would open up a completely new targeting space for therapeutic antibodies.

## Methods

### Cell culture

The LS174T, MKN45 and colo320HSR cell lines, which are adherent in culture, were grown at 37 °C in a humidified 5% CO_2_ atmosphere in RPMI medium 1640 + Glutamax (Gibco) supplemented with 10% inactivated fetal calf serum. The FreeStyle™ HEK293FS cell line was grown in suspension at 37 °C in a humidified 5% CO_2_ atmosphere with 115 rpm agitation in Freestyle™ 293 expression medium with Glutamax (Gibco) medium supplemented with 1 mM sodium pyruvate (Gibco), 2 mM glutamine (Gibco), 100 U/ml penicillin and 100 µg/ml streptomycin (Gibco).

### Generation and purification of antibodies

Parental anti-CEACAM5 antibody sequence was available from earlier in-house work, where it had been obtained using conventional mouse immunization and hybridoma technology, and humanized afterwards. The protein sequences of the antibody light and heavy chain is depicted below, with CDR indicated in **bold** and constant region in *italic*:

Anti-CEACAM5_light_chain:

DIQMTQSPASLSASVGDRVTITCRAS**ENIFSY**LAWYQQKPGKSPKLLVY**NTR**TLAEGVPSRFSGSGSGTDFSLTISSLQPEDFATYYC**QHHYGTPFT**FGSGTKLEIK*RTVAAPSVFIFPPSDEQLKSGTASVVCLLNNFYPREAKVQWKVDNALQSGNSQESVTEQDSKDSTYSLSSTLTLSKADYEKHKVYACEVTHQGLSSPVTKSFNRGEC*.

Anti-CEACAM5_heavy_chain: EVQLQESGPGLVKPGGSLSLSCAAS**GFVFSSYD**MSWVRQTPERGLEWVAY**ISSGGGIT**YAPSTVKGRFTVSRDNAKNTLYLQMNSLTSEDTAVYYC**AAHYFGSSGPFAY**WGQGTLVTVSS*ASTKGPSVFPLAPSSKSTSGGTAALGCLVKDYFPEPVTVSWNSGALTSGVHTFPAVLQSSGLYSLSSVVTVPSSSLGTQTYICNVNHKPSNTKVDKKVEPKSCDKTHTCPPCPAPELLGGPSVFLFPPKPKDTLMISRTPEVTCVVVDVSHEDPEVKFNWYVDGVEVHNAKTKPREEQYNSTYRVVSVLTVLHQDWLNGKEYKCKVSNKALPAPIEKTISKAKGQPREPQVYTLPPSRDELTKNQVSLTCLVKGFYPSDIAVEWESNGQPENNYKTTPPVLDSDGSFFLYSKLTVDKSRWQQGNVFSCSVMHEALHNHYTQKSLSLSPG*.

Nucleic acid sequences coding for the antibody heavy or light chains were cloned into mammalian expression plasmids under the CMV enhancer/promoter and the SV40 polyA signal. Resulting plasmids were transfected into FreeStyle™ HEK293 cells (Thermo Fisher Scientific; K9000-10) using FreeStyle™ 293 Expression System according to the manufacturer’s instructions. Antibodies were purified by protein A affinity chromatography, desalted on mini trap Sephadex G-25 column, sterilized with membrane filter (Millex®GC, 0.22 µm) and stored in PBS. The concentrations were determined using Dropsense (PerkinElmer) using the molar extinction coefficient calculated from the sequence.

### Antibody characterization

SEC-HPLC was used to analyze the purity of the antibodies after the purification process. Protein electrophoresis under reduced and non-reduced conditions were performed using the 2100 Bioanalyzer System (Agilent). A reverse phase liquid chromatography mass spectrometry (LC-MS) was carried out using a Qtof premier instrument (Waters). All antibodies were diluted in PBS at 1 mg/ml and mixed with DTT at a final concentration of 0.2 µM for 30 min at 37 °C under agitation. Fifteen µg of reduced samples were loaded on a Jupiter C4 column (150 × 2 mm, Phenomenex) and eluted at a flow rate of 0.35 ml/min using a step gradient of 50% of B after 11.9 minutes (mobile phase A: 0.03% of TFA in water and mobile phase B: 0.03% of TFA in acetonitrile). Peaks were assigned based on their expected molecular mass.

### Surface plasmon resonance

Sierra Sensors MASS-2 instrument and Biacore T200 instruments were used for the kinetic studies. Anti-human Fc surfaces were prepared by covalently immobilizing anti-human Fc antibody (Human antibody capture kit, Amine coupling kit, GE LifeSciences) on HCA or CM5 surfaces respectively. Briefly, the surfaces were activated with a 7 min pulse of EDC/NHS mixture. The anti-human Fc antibody was diluted to 25 µg/ml in 10 mM sodium acetate pH5.0 and injected over the activated surfaces for 7 min. The surfaces were deactivated with a 7 min pulse of 1 M Ethanolamine, pH8.5.

Antibodies were diluted at 1 µg/ml in HBS-EP + buffer (GE LifeSciences) and captured for 1 min at 10 µl/min. As antigen, we used the extracellular domain of human CEACAM5 produced in mammalian cells with a C-terminal His-tag and purified by Nickel affinity chromatography. Antigen concentration series starting from 300 nM was prepared in HBS-EP + buffer and injected for 4 min at 30 µl/min. Dissociation was monitored for 15 min. Surfaces were regenerated with a 30 s pulse of 3 M MgCl_2_. All experiments were run with HBS-EP + buffer. Sensorgrams were double-referenced by subtracting blank injections and reference flow cells. Data were fitted with simple 1:1 binding model using either Sierra analyzer or Biacore T200 Evaluation softwares. On/Off rate plots were drawn in GraphPadPrism software.

### Protein thermal stability measurements

To compare the stability of CPP-Abs and Ab-ctrl2, thermal unfolding profiles of the proteins were recorded by nano-DSF using a Prometheus instrument. For this purpose, 10 µl of a 1 mg/ml solution of each antibody in PBS buffer were loaded into nanoDSF grade standard capillaries. Thermal unfolding was analyzed in a thermal ramp from 25 °C to 80 °C with a heating rate of 1 °C/min. Unfolding transition temperatures (Tm) were automatically determined by the software (PR.thermcontrol).

### Cellular binding assay

LS174T and MKN45 cells were distributed in 96-well plates (10^5^ cells per well). The different antibody controls and CPP-Abs were incubated at 10 µg/ml in 100 µl of PBS BSA 1% during 20 min at 4 °C. Cells were then washed twice with PBS 1% BSA and a secondary antibody Alexa Fluor®488 AffiniPure Goat Anti-human IgG Fc gamma specific (Jackson) was added at a dilution of 1/400, prior to 15 min incubation at 4 °C. Cells were then washed twice and resuspended in PBS BSA 1% in 150 µl for FACS analysis.

### Split GFP complementation assay

To quantify the amount of antibodies that reaches the cytosol, we implemented a simple and direct readout based on split GFP fluorescence complementation assay. When the large fragment of GFP, GFP1-10, reaches the small fragment (GFP11) fused at the C-terminal of the heavy chain, the 2 GFP fragments complement and GFP fluorescence can be measured. To increase the sensitivity of the system, we added SBP2 (streptavidin binding protein 2) and streptavidin (SA) to GFP11 and GFP1-10 respectively^32,42^. For cell lysate complementation and electroporation assay, HEK293FS cells were transfected with GFP1-10SA- expressing plasmid using 293fectin^TM^ transfection reagent (Gibco) according to manufacturer’s instructions. For cell lysate experiments, cells were lysed 48 hours after transfection in a PBS buffer with 0,01% of Triton X-100 (VWR) and protease inhibitors (cOmplete^TM^, mini EDTA-free protease inhibitor cocktail) for 1 h at 4 °C under agitation. Cell lysates were assayed using BCA protein kit (Coomassie®plus protein Assay reagent, Pierce) and diluted to a concentration of 0.6 mg/ml. This diluted lysate was distributed in a black well-plate and 50, 5 or 0.5 µg of antibodies was added. Fluorescence was followed by spectrofluorimetry (Infinite®200PRO, TECAN). For electroporation, 10^7^ cells were concentrated into cassettes with 100 µg of each antibody construct. Cells were electroporated using HEK293 cells protocol of MaxCyte STX® electroporation system and seeded back into 50 ml Erlenmeyers. Cells were incubated 40 min without rotation at 37 °C in a humidified 5% CO_2_ atmosphere, then diluted with 10 ml of medium and incubated with agitation for 8 hours before cytometry analysis.

### Cell penetration assay

Cells were transfected 24 hours post-seeding with the plasmid expressing GFP1-10SA in 24 well-plate. For LS174T and colo320HSR cells, plasmids were transfected using Fugene®HD transfection reagent (Promega) according to manufacturer’s instructions. For MKN45, 293fectin^TM^ transfection reagent (Gibco) was used to transfect GFP1-10SA plasmids into cells. Antibodies were added 24 hours after transfection and cells were further incubated for 24 hours. Cells were treated with TryPLE Select (Gibco) for 5 min, collected in 96 well-plate, washed with PBS and treated with ebioscience^TM^ Fixable viability Dye eFluor^TM^ 780 (Invitrogen) to stain dead cells during 20 min at 4 °C. Cells were washed and resuspended in PBS 1% BSA before FACS analysis.

### Quantification of cytosolic antibody

We correlated fluorescence intensity with the number of GFP molecules by FACS analysis of beads coated with different and known amounts of GFP. Use of this standard allows determining the number of antibodies in the cytosol from the fluorescence intensity in the cells. To evaluate the fluorescent intensity resulting from the antibody inside the cytosol we subtracted the cells´ autofluorescence. To take into account the relative brightness of split versus intact GFP, we applied a factor of 1.65, as split GFP is less bright than GFP^32^. Assuming the cytosolic volume of LS174T cells of 940 µm^3^, we evaluated the concentration of cytosolic antibodies with the following formula:

$${\rm{nM}}/{\rm{cell}}=\frac{{\rm{number}}\,{\rm{of}}\,{\rm{complemented}}\,{\rm{split}}\,{\rm{GFP}}\,{\rm{molecules}}\,{\rm{per}}\,{\rm{cell}}\ast 1.65}{{\rm{Avogadro}}\,{\rm{constant}}\ast 940\ast {10}^{15}}$$

### Flow cytometry

Cells were processed on a CytoFLEX (Beckman Coulter’s) flow cytometer and results analyzed with FlowJo X 10.0.7 (Tree Star Inc). GFP was analyzed using FITC channel (Excitation: 495 nm, emission: 521 nm) and live/dead cells with APC channel (Excitation: 650 nm, emission: 660 nm). Gating of the single cell population (distributed FSC-H vs FSC-W) was followed by gating of the viable single cell population according to live/dead stain status, and this population was further divided into fluorescent (FITC+) and not fluorescent (FITC−). For cell viability study, all cells were studied and gated with the live/dead stain status.

### Statistics

Data are represented as the mean ± sd of 3 independent experiments. A two-way ANOVA with the Fisher’s multiple comparison tests was used to determine significance between samples. The reference sample is specified in figure legends.

## Supplementary information

Supplementary Tables and Figures

## Data Availability

All data generated or analysed during this study are included in this published article (and its Supplementary Information files).
