# Supplementary material for: Intracellular delivery of therapeutic antibodies into specific cells using antibody-peptide fusions
Source: Sci Rep. 2019 Dec 10;9:18688. doi: 10.1038/s41598-019-55091-0 (PMC6904672; doi:10.1038/s41598-019-55091-0)
Supplement: Supplementary file 1 — Supplementary Tables and Figures [file 41598_2019_55091_MOESM1_ESM.pdf]

SUPPLEMENTARY MATERIAL

Intracellular delivery of therapeutic antibodies into specific cells using antibody-peptide fusions

Julie Gaston[1], Nicolas Maestrali[2], Guilhem Lalle[3], Marie Gagnaire[2], Alessandro Masiero[2], Bruno Dumas[2], Tarik Dabdoubi[2], Katarina Radošević[2]*, Pierre-François Berne[1]

[1]Yubsis, 4 rue Pierre Fontaine, 91000 Evry, France

[2]Sanofi R&D, Biologics Research, 13 Quai Jules Guesde, 94400 Vitry-sur-Seine, France

[3]Department of Immunology, Virology and Inflammation, UMR INSERM 1052, CNRS 5286, Centre Léon Bérard, Labex DEVweCAN, 693743 Lyon, France

*corresponding author: Katarina Radošević, katarina.radosevic@sanofi.com

Supplementary Tables

Supplementary Table S1: Affinity of CPP-Abs

Name	ka [1/(M·s)]	kd [1/s]	KD [M]	KD ratio (/WT)
Pep-1-AH	1.82E+05	3.03E-03	1.66E-08	0.9
Pep-1-BH	1.24E+05	3.54E-03	2.85E-08	1.5
Pep-1-LCC	1.32E+05	3.09E-03	2.34E-08	1.3
Ab-ctrl1	2.38E+05	2.89E-03	1.22E-08	1.0

ka: association rate; kd: dissociation rate; KD: equilibrium constant.

Supplementary Table S2: Affinity of CPP-Abs with cytosolic tag

Name	ka [1/(M·s)]	kd [1/s]	KD [M]	KD ratio (/WT)
Ab-ctrl2	3,95E+05	4,19E-03	1,05E-08	0,8
PEPth-LCC	2,95E+05	6,24E-03	2,12E-08	1,5
PEPth-BH	2,93E+05	6,39E-03	2,18E-08	1,6
PEPth-AH	2,97E+05	5,76E-03	1,94E-08	1,4
Pep-1-LCC	2,91E+05	5,48E-03	1,88E-08	1,4
Pep-1-BH	3,23E+05	7,43E-03	2,30E-08	1,7
Pep-1-AH	3,68E+05	6,62E-03	1,80E-08	1,3
aurein-LCC	3,02E+05	3,48E-03	1,15E-08	0,9
aurein-BH	2,00E+05	3,91E-03	1,96E-08	1,6
aurein-AH	2,27E+05	3,43E-03	1,51E-08	1,2
MTS-LCC	3,15E+05	3,29E-03	1,05E-08	0,9
MTS-BH	3,47E+05	3,52E-03	1,01E-08	0,8
MTS-AH	4,28E+05	3,00E-03	7,00E-09	0,6
GFWFG-LCC	nd	nd	nd	nd
GFWFG-BH	3,61E+05	2,97E-03	8,23E-09	0,7
GFWFG-AH	3,31E+05	3,10E-03	9,39E-09	0,8

ka: association rate; kd: dissociation rate; KD: equilibrium constant. nd: not determined.
GFWFG-LCC was not efficiently captured on surface and the binding to CEACAM5 could not be determined.

Supplementary Table S3: MFI values corresponding to the number of GFP molecules coated on beads (using calibration beads)

peaks	B	C	D	E	F	G
MESF	74471	164770	410977	1291440	7372326	18463961
MFI (x100)	0,52	1,45	5,52	20,56	119,07	353,61

MESF: Molecular Equivalent of Soluble Fluorophore

Supplementary Figures

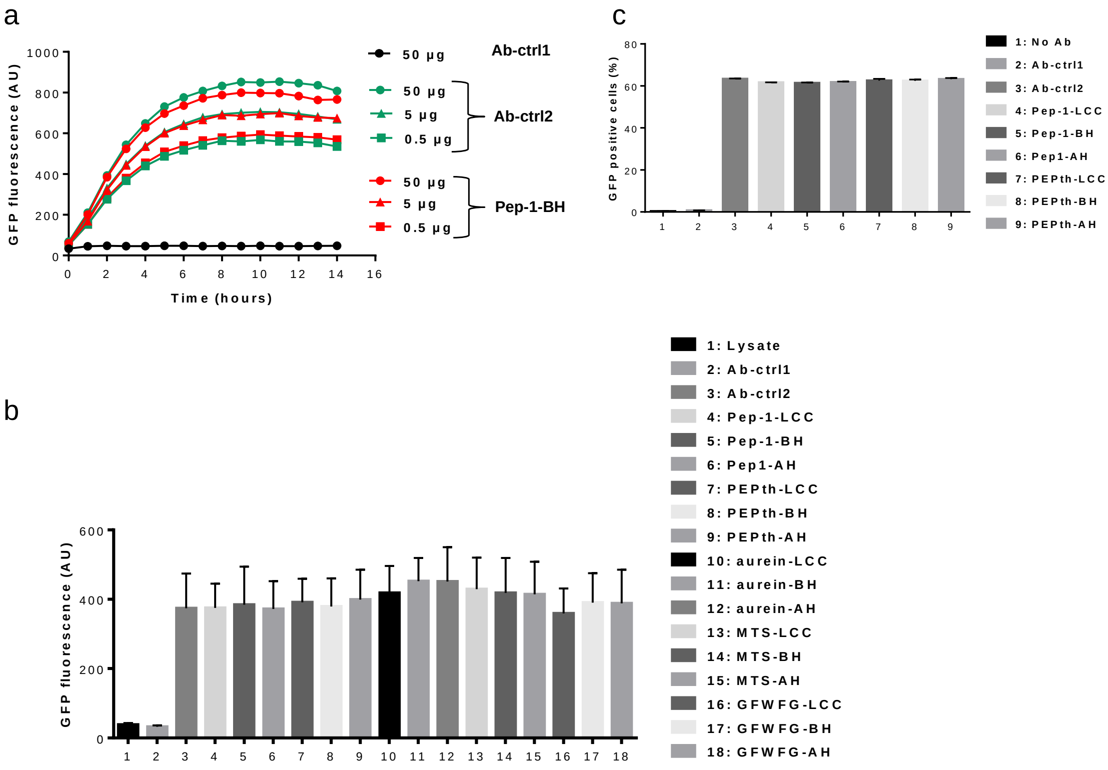

Supplementary Figure S1. Validation of split GFP complementation assay. Fixed amount of HEK293FS cell lysate expressing GFP1-10SA was incubated with different amounts of antibodies (0.5, 5 or 50 µg). Ab-ctrl1 without the cyto-Tag, Ab-ctrl2, which carries the GFP11-SBP2 cyto-Tag at C-terminus of the HC, and Pep-1-BH were evaluated for their capacity to complement with GFP1-10SA. Fluorescence is analyzed by spectrofluorimetry (**a**). All the CPP-Abs were incubated at 0.5 µg in HEK293FS cell lysate expressing GFP1-10SA for 8 hours and fluorescence intensity was analyzed by spectrofluorimetry (**b**). HEK293FS cells were transiently transfected with GFP1-10SA (transfection efficiency ~90%) and after 48 hours were electroporated to force antibodies delivery into the cytosol. Percentage of GFP-positive cells was analyzed by FACS (**c**).

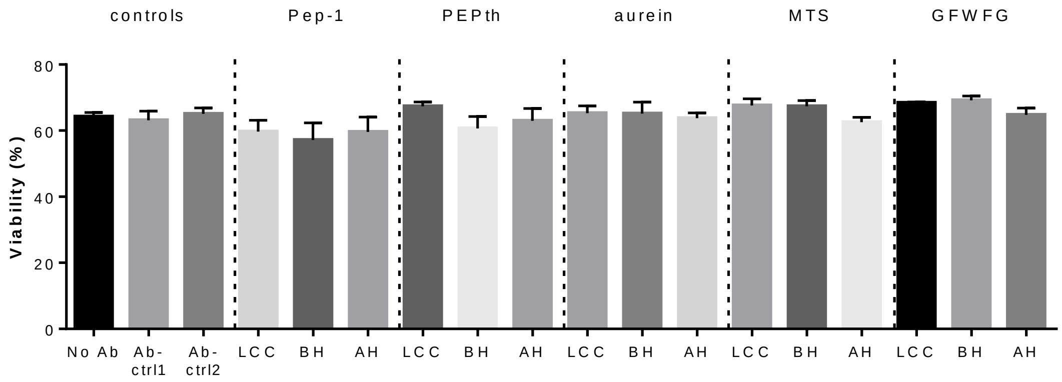

Supplementary Figure S2. Cell viability analysis. Cell viability after transfection and incubation with cpAbs is calculated by FACS using cell viability marker on all cells.

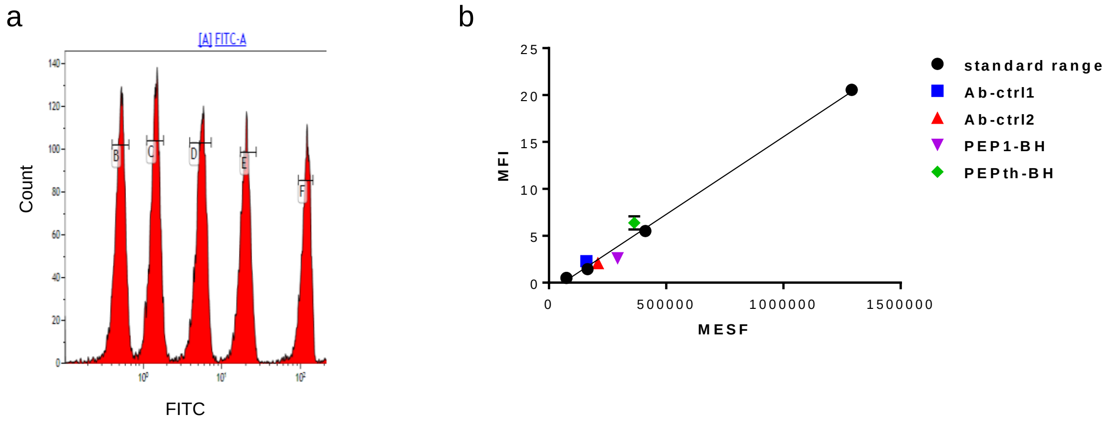

Supplementary Figure S3. Histogram showing the fluorescence of GFP-coated beads. Five different intensities (from B to F) which represent 5 different known amounts of coated GFP (**a**). Standard plot for GFP fluorescence showing linear regression of the amount of GFP coated on beads versus the GFP fluorescence intensities R2= 0.999. Results with 5 µM samples were added (**b**).
